# Impaired Binocular Depth Perception in First-Episode Drug-Naive Patients With Schizophrenia

**DOI:** 10.3389/fpsyg.2018.00850

**Published:** 2018-05-31

**Authors:** Zhengchun Wang, Zhipeng Yu, Zhichao Pan, Keyu Zhao, Qiqi Zhao, Dongsheng Zhou, Hao-Wei Shen, Xiangping Wu

**Affiliations:** ^1^School of Medicine, Ningbo University, Ningbo, China; ^2^Ningbo Kangning Hospital, Ningbo, China

**Keywords:** binocular depth perception, drug-naive, first episode, schizophrenia, visual processing

## Abstract

Binocular depth perception (BDP) is one of the most demanding visual function that involves both dorsal and ventral visual information streams. Substantial research has been conducted on the disruption of BDP in patients with schizophrenia. However, research on first-episode and drug-naive patients with schizophrenia (FEDN) is limited. To assess the BDP of schizophrenia patients while controlling for the effects of antipsychotics and the duration of illness. We investigated BDP in patients with schizophrenia via the Titmus Stereopsis Test in this study, by matching the patients into three groups: FEDN (*n* = 17), long duration of illness and medicine treatment (LDMT) (*n* = 31) and the healthy control group (*n* = 40). Results showed that both the FEDN (mean = 1.71, 95% confidence interval [CI]: [1.57, 1.84]) and LDMT (1.73, 95% CI: [1.66, 1.81]) patients displayed a significant decline (*p* = 0.01, Cohen’s *d* = 0.67, *p* = 0.001, Cohen’s *d* = 0.92, respectively) in depth perception compared to the healthy control (1.55, 95% CI: [1.48, 1.61]) group. Additionally, there were no significant differences (*p* = 0.68, Cohen’s *d* = 0.11) between the FEDN and LDMT groups, and no correlation (Pearson r = -0.16, *p* = 0.38, *R*^2^ = 0.03) was observed between the duration of illness and impaired BDP in the LDMT group. The proportion of individuals with stereopsis detection in either FEDN (12/17) or LDMT (26/31) groups under stereo threshold 63 arc seconds (″), were significantly lower (Pearson χ^2^ = 6.29, *p* = 0.043, φ_c_ = 0.27) compared to the healthy control group (38/40). Significant difference in stereopsis detection also occurred at 50″ (Pearson χ^2^ = 12.31, *p* = 0.001, φ_c_ = 0.37), 40″ (Pearson χ^2^ = 12.38, *p* = 0.002, φ_c_ = 0.38), 32″ (Pearson χ^2^ = 6.69, *p* = 0.035, φ_c_ = 0.28), 25″ (Pearson χ^2^ = 14.82, *p* = 0.001, φ_c_ = 0.41) and 20″ (Pearson χ^2^ = 6.73, *p* = 0.034, φ_c_ = 0.28) between the three groups. These findings showed a moderately strong association between schizophrenia and defective stereopsis.

## Introduction

Patients with schizophrenia is associated with a widespread deficiency in cognitive coordination. Perceptual processing has been recognized as an important domain in the assessment of cognitive function of patients with schizophrenia (Butler et al., 2008; Javitt, 2009). Visual sensory processing deficits are prevalent among individuals with schizophrenia (Silverstein and Keane, 2011b; Skottun and Skoyles, 2013; Yoon et al., 2013; Silverstein and Thompson, 2015; Silverstein et al., 2015), including impairments in contrast sensitivity (Kiss et al., 2010; Kelemen et al., 2013), forward and backward masking (Green et al., 2011), surround suppression (Dakin et al., 2005; Yoon et al., 2010), perceptual organization (Silverstein and Keane, 2011a), form processing (Javitt, 2009), and motion processing (Chen, 2011). Therefore, investigation into the visual sensory processing in schizophrenia can accelerate our understanding of schizophrenia-related information processing impairment (Silverstein and Keane, 2011b; Yoon et al., 2013; Silverstein et al., 2015).

Binocular depth perception (BDP) is a critical function of the early visual system, which is derived by the difference between the images impressed upon the left and right retina created by the distance between the eyes (∼60 mm) (Parker, 2007). Previous literatures have showed that the averaged depth perception was lower in schizophrenics and that the median threshold for stereopsis is higher. Impaired BDP was found in patients with schizophrenia using the Graded Circle test (Schechter et al., 2006; Hui et al., 2017), the Frisby Stereo Test and random dot stereograms (Kantrowitz et al., 2009). The dysfunctional BDP was also found in schizotypal personality traits (SPT) (Barbato et al., 2012) and individuals at clinical high risk (CHR) of developing psychosis (Lee et al., 2012), although the dysfunction was less severe compared to patients with schizophrenia. Notably, a previous study showed that young subjects with clinically high risk of developing schizophrenia (CHR) had normal stereopsis compared with healthy controls. The preliminary evidence suggests that BDP deficit might correlate with schizophrenia.

Previous studies found that there is no association between impaired depth perception in patients with schizophrenia and positive and/or negative symptoms or antipsychotic medications (Schechter et al., 2006; Kantrowitz et al., 2009). These findings implied that stereopsis dysfunction might be steady in a different state of schizophrenia. It is worth noting that antipsychotic drugs and duration of the disease have been found to affect visual contrast sensitivity dysfunctions in patients with schizophrenia (Shoshina et al., 2014; Shoshina and Shelepin, 2015). Therefore, whether antipsychotics or duration of schizophrenia adversely affect BDP needs to be assessed with direct evidence. As far as we know, stereopsis of the first-episode and drug-naive patients with schizophrenia (FEDN) has not yet been investigated in previous studies. We anticipated that each patient group’s stereo acuity will be lower than the controls but not differ from each other.

In the current study, a standardized stereoscopic depth perception test was performed to examine BDP in first-episode and drug-free patients with schizophrenia, and patients with chronic schizophrenia under age- and sex-matched healthy controls. Our findings would add further weight to the proposal that BDP deficits might be involved in the spectrum of neurological changes and that impaired BDP was associated with patients with schizophrenia.

## Materials and Methods

This research has been approved by the ethics committee of Ningbo Kangning Hospital, and was conducted as per the guidelines of the Declaration of Helsinki. All participants were provided with a written informed consent and were able to take part in stereopsis assessment. Each subject gave informed consent and that patient anonymity has been preserved.

### Participants

The study comprised of 48 patients with schizophrenia and 40 healthy controls (18 male, 22 female). Basic demographic information such as age, gender, education, corrected vision, medical history, physical and psychotic examination was collected from patients with schizophrenia and healthy controls (**Table 1**).

**Table 1:** Demographic and clinical characteristics of FEDN, LDMT schizophrenia and healthy controls.

	FEDN schizophrenia	LDMT schizophrenia	Healthy controls
Sample number	*n* = 17	*n* = 31	*n* = 40
Gender (male/female)	8/9	19/12	18/22
Age (years)	32.29 ± 1.96	32.77 ± 0.74	30.65 ± 1.28
Education (years)	10.47 ± 0.66	11.94 ± 0.34	12.32 ± 0.47
Left eye (logMAR)	0.004 ± 0.014	0.003 ± 0.073	0.025 ± 0.098
Right eye (logMAR)	0.009 ± 0.022	0.004 ± 0.090	0.013 ± 0.106
Illness onset (years)		22.81 ± 0.83	
Duration of illness (years)		9.97 ± 0.74	

*FEDN and LDMT indicates first-episode and drug-naïve and long duration of illness and medicine treatment, respectively. MAR indicates the minimum angle of resolution. Mean ± SEM.*

Seventeen of the schizophrenic were antipsychotic-naive patients of the first psychotic episode (8 males, 9 females), 31 were long-term patients (>12 months) receiving antipsychotic treatment (19 males, 12 females). Patients were recruited from the inpatient and outpatient unit at Ningbo Kangning Hospital of Zhejiang Province. Patients’ diagnosis was made by two independent psychiatrists. All participants met the DSM–V/ICD-10 diagnostic criteria for schizophrenia.

The inclusion criteria for the first-episode drug-naive schizophrenia group (FEDN) was similar to a previous study (Dai et al., 2018): (1) the first acute episode that met DSM-V/ICD-10 criteria for schizophrenia; (2) duration of symptoms not longer than 2 years; (3) no prior treatment with antipsychotic medication; (4) aged 18–45 years; (5) received education for at least 6 years. The inclusion criteria for schizophrenia in the long duration of illness and medicine treatment (LDMT) group were: (1) >1-year illness duration; (2) received a stable dose of oral antipsychotics for at least 6 months before entry into this study; (3) aged 18–45 years; (4) received education for at least 6 years. Healthy controls were recruited from Ningbo Kangning Hospital and Ningbo University. None of them presented a personal or family history of psychiatric disorder. Any subjects with a history of medical illnesses or drug and alcohol abuse/dependence were excluded.

The eligible subjects had to meet the following BDP requirements: (1) vision acuity of left and right eyes reached at least 0.6 after correction respectively, (2) the acuity discrepancies between two eyes were no more than 1 line of E Standard Logarithm Eyesight Table, and (3) no history of visual or ocular pathology. Visual acuity (VA) is measured according to the size of letters viewed on a Snellen chart, expressed as the logarithm of the minimum angle of resolution (logMAR), and is used as the scale in the current study.

### Binocular Depth Perception Measures

Before the examination, test procedures and aims were explained carefully and clearly to each of the subjects. A few easy tests (low stereo threshold) were conducted to ensure that each participant, especially patients with schizophrenia, could understand and perform the test accurately.

Binocular depth perception was assessed by measuring stereo acuity under natural light via the Titmus Stereopsis Test (Stereo Optical Co., Chicago, IL, United States), which includes tests that were used in previous studies with schizophrenia patients (Schechter et al., 2006; Kantrowitz et al., 2009; Hui et al., 2017). The Titmus Stereopsis Test consists of three sections: Random Dot Stereogram (RDS), Animals Stereo test and the Graded Circle Stereo test (GCS). The GCS provides a finely graded sequence for critical testing that was designed mainly for adults. The GCS consists of nine groups of circles. Each group contains four circles, and only one of these circles (the target) has a degree of crossed disparity. The target appears to be perceptually closer to subjects than other circles, or is floating above the plane of the other three circles when viewed through polarized glasses. The crossed disparity ranges from large (400″) to small (25″), making the test progressively more difficult. It is a Yes/No test and is measured in visual arc seconds (″). Participants were requested to indicate whether there was one circle that stood out from the remaining three circles.

### Statistical Analysis

The differences between patients with schizophrenia and healthy controls were compared using ANOVA for VA, age, education and VA, and χ^2^ test for gender. Stereo acuity threshold is defined as the minimal angle of stereopsis observed when the participant responds correctly. A One-sample Kolmogorov–Smirnov test was used to examine whether the data has a normal distribution, and the result indicated that the raw data of healthy controls was in a normal distribution (*p* = 0.511), while the raw data of patients groups (FEDN and LTDM) did not follow a normal distribution (*p* = 0.005, *p* = 0.016, respectively). Stereo acuity thresholds were log transformed for normality prior to the following analysis. Stereo acuity was analyzed using one-way ANOVA followed by Bonferroni *post hoc* tests for multiple comparisons. The relationship among measures was determined by Pearson correlations and linear regressions. Subjects responding (detected stereo or not) at each angle of stereopsis was analyzed using the χ^2^. All data are shown as 95% confidence levels (CI) and standard error of the mean (SEM). All comparisons were two-tailed with a significance level of 5% and performed by SPSS 17.0 (SPSS Inc., Chicago, IL, United States). Additionally, the effect sizes were calculated for each kind of statistics (Lenhard and Lenhard, 2016).

## Results

Basic demographic information such as gender, age, education and VA were collected for the first-episode drug-naive schizophrenia (FEDN), schizophrenia with LDMT and healthy controls groups (**Table 1**). No significant differences between the groups were observed in gender (Pearson χ^2^= 1.99, *p* = 0.37, φ_c_ = 0.15), age [*F*_(2,87)_ = 2.47, *p* = 0.09] and education [*F*_(2,87)_ = 2.74, *p* = 0.07]. The average age of illness onset and illness duration in LDMT schizophrenia were 22.81 ± 0.83 and 9.97 ± 0.74 years. All participants had normal or corrected-to-normal VA and there was no significant difference [*F*(5,175) = 0.31, *p* = 0.91, $\eta_{p}^{2}$ = 0.01] in the VA for the three test groups.

There were no statistical outliers within the stereo thresholds of the FEDN and LDMT groups according to the mean ± 3σ criterion, while there was one value (160″) that was extremely high (mean + 3σ = 114.71″) in the healthy control group. The stereo thresholds of the healthy controls were in a normal distribution (*p* = 0.511, One-sample Kolmogorov–Smirnov test), while the stereo thresholds of the patients groups (FEDN and LDMT) did not follow a normal distribution (*p* = 0.005, *p* = 0.016, respectively, One-sample Kolmogorov–Smirnov test), therefore, all raw stereo thresholds were log transformed to make it normal. The log transformed stereo values of FEDN, LDMT and healthy control groups followed a normal distribution (*p* = 0.79, *p* = 0.13, and *p* = 0.35, respectively, One-sample Kolmogorov–Smirnov test) and there were no statistical outliers within any of the three groups according to the mean ± 3σ criterion. Both the FEDN and LDMT groups had a median stereo acuity threshold of 1.70 (i.e., 50″), while the healthy controls had a threshold of 1.51 (i.e., 32″). One-way analysis of the Graded Circles Stereo Test showed significant differences between the FEDN, LDMT and healthy control groups [*F*_(2,87)_ = 7.27, *p* = 0.001, $\eta_{p}^{2}$ = 0.15].

**FIGURE 1:**
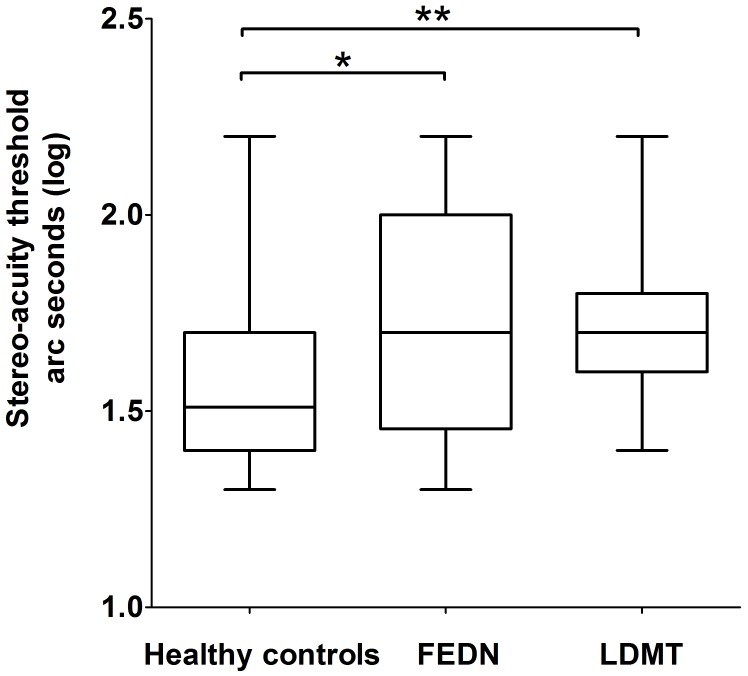
Difference in binocular depth perception stereo acuity (log seconds of arc) between healthy controls, first-episode and drug-naive patients of schizophrenia (FEDN) and long duration of illness and medicine treatment (LDMT). ^∗^Indicates *P* < 0.05; ^∗∗^indicates *P* < 0.01.

Further *post hoc* analysis on the log transformed stereo threshold revealed that FEDN and LDMT schizophrenias, displayed a significant reduction in sensitivity to stereopsis compared to the healthy controls (mean ± SEM = 1.55 ± 0.03, median = 1.51, 95% CI: [1.30, 2.20]; *p* = 0.01, *p* = 0.001, respectively; **Figure 1**). However, no significant difference was observed (*p* = 0.68) between the FEDN (mean ± SEM = 1.71 ± 0.07, median = 1.70, 95% CI: [1.30, 2.20]) and LDMT groups (mean ± SEM = 1.73 ± 0.04, median = 1.70, 95% CI: [1.40, 2.20]).

**FIGURE 2:**
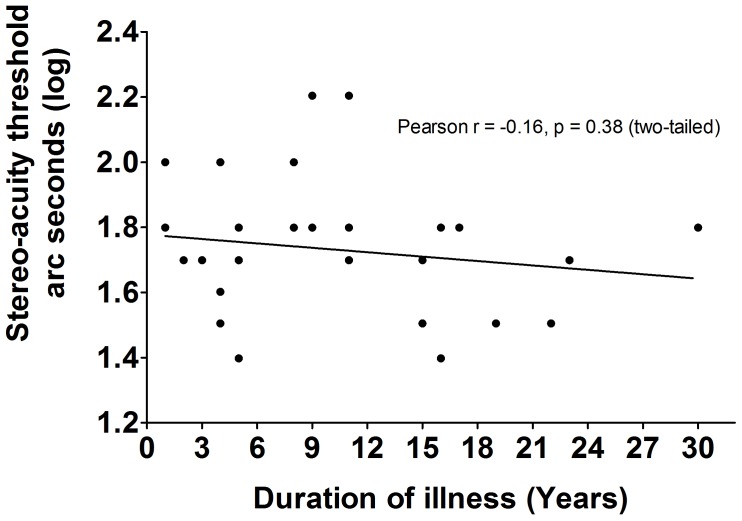
The correlations between duration of illness and BDP in LDMT schizophrenia. There were no significant correlations (Pearson *r* = –0.16, *P* = 0.38, *R*^2^ = 0.03) between duration of illness and BDP in LDMT schizophrenia.

The LDMT schizophrenia group was divided according to disease durations. Log transformed stereo acuity in patients with schizophrenia of less than 10 years (*n* = 17, mean ± SEM = 1.76 ± 0.049), was similar [*t*_(29)_ = -0.97, *p* = 0.34, *d_Cohen_* = 0.35] to patients suffering schizophrenia for more than 10 years (*n* = 14, mean ± SEM = 1.69 ± 0.055). These results were confirmed by determining that there were no correlations between the duration of illness and the log transformed seconds of arc in patients with schizophrenia (Pearson *r* = -0.16, *p* = 0.38, *R*^2^= 0.03; **Figure 2**). Although the effect size were small due to similar patients’ results with various disease duration, there was no effect of disease duration or medication based on the statistical analysis.

**FIGURE 3:**
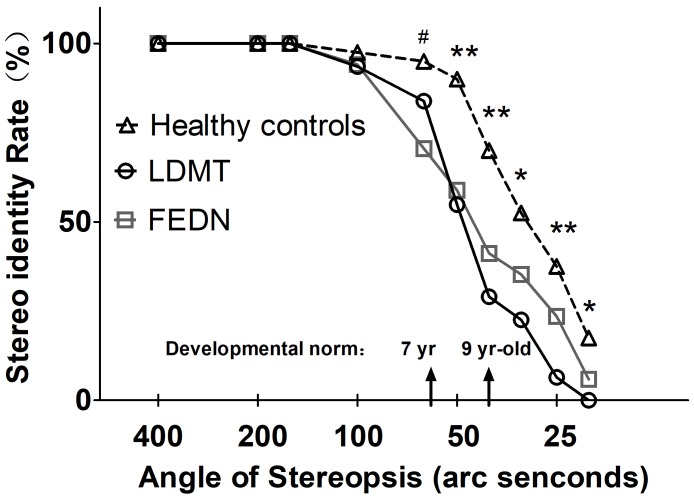
Stereopsis detection at indicated angles of stereopsis for FEDN, LDMT and healthy control groups. Smaller angle of stereopsis correspond to increased task difficulty. ^∗^Indicates difference between healthy controls and LDMT *P* < 0.05; ^∗∗^Indicates difference between healthy controls and LDMT *P* < 0.01; ^#^Indicates difference between healthy controls and FEDN *P* < 0.01.

**FIGURE 4:**
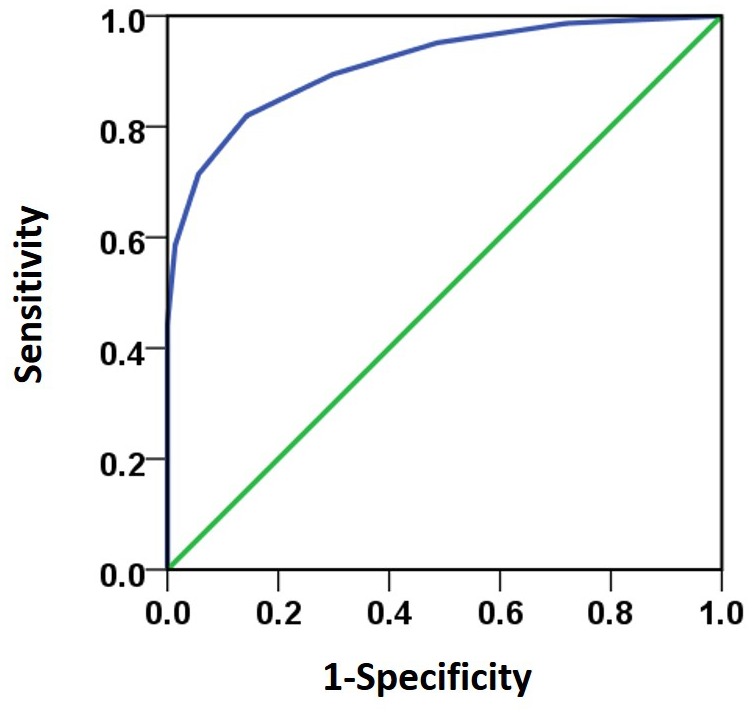
The ROC curve analyses was used to analyze subjects’ response to various stereo thresholds. The AUC (0.91) was significant higher (*p* = 0.0001) than the 0.5 level. The sensitivity and specificity at the optimal critical point were 0.82 and 9.86, respectively.

Besides the stereo threshold, we also examined the stereopsis detection of the three groups during the stereoscopic depth perception test. To assess whether there were differences among the three groups at each angle of stereopsis, the χ^2^ test were used. The results showed that the stereopsis detection of the three groups were significantly different at 63″ (Pearson χ^2^ = 6.29, *p* = 0.043, φ_c_ = 0.27), 50″ (Pearson χ^2^ = 12.31, *p* = 0.001, φ_c_ = 0.37), 40″ (Pearson χ^2^ = 12.38, *p* = 0.002, φ_c_ = 0.38), 32″ (Pearson χ^2^ = 6.69, *p* = 0.035, φ_c_ = 0.28), 25″ (Pearson χ^2^ = 14.82, *p* = 0.001, φ_c_ = 0.41) and 20″ (Pearson χ^2^ = 6.73, *p* = 0.034, φ_c_ = 0.28) between the three groups (**Figure 3**). The exact values were listed in **Table 2**, which provided the distribution of the “Titmus stereo thresholds” of each group and might give an indication of the potential usefulness of impaired stereopsis as a measure of discriminability of the circle stereopsis test. The Receiver Operating Characteristic (ROC) curve analyses showed that the area under curve (AUC) was 0.91, *p* = 0.0001. The sensitivity and specificity at the optimal critical point were 0.82 and 9.86, respectively (**Figure 4**).

**Table 2:** Number of subjects that detected (“1”) or did not detect (“0”) the stereo pattern at each angle of stereopsis in the FEDN, LDMT and healthy control groups.

Groups	Detection	400″	200″	160″	100″	63″	50″	40″	32″	25″	20″
FEDN	1	17	17	17	16	12	10	7	6	4	1
	0	0	0	0	1	5	1	10	11	13	16
LDMT	1	31	31	31	29	26	17	9	7	2	0
	0	0	0	0	2	5	14	22	24	29	31
Healthy Control	1	40	40	40	39	38	36	28	21	15	7
	0	0	0	0	1	2	4	12	19	25	33

*FEDN and LDMT indicates first-episode and drug-naïve and long duration of illness and medicine treatment, respectively.*

To check for possible confounds in the results due to VA and optical correction, we examined BDP on another group of healthy controls who was wearing glasses (*n* = 14) or not (*n* = 12) during the test and examined the relationship between the ocular diopter strength and the BDP threshold. We found no difference [*F*(24) = 0.99, *p* = 0.67] in the BDP between subjects wearing glasses (mean ± SEM = 36.14 ± 9.77, 95% CI: [15.03, 57.26]) or not (31.41 ± 3.65, 95% CI: [23.39, 39.44]) and no association (Pearson *r* = -0.26, *p* = 0.36) between the ocular diopter strength and the BDP threshold was found.

## Discussion

The experimental results showed that the power to detect differences according to group or seriousness or length of disease was low, while there are consistent with our prediction, both the FEDN and LDMT groups suffered from impaired BDP with similar severity. The threshold of stereo-acuity in the two patient groups was increased by 16 and 18″ respectively, compared to the healthy controls. In the LDMT group, the severity of BDP impairment had no correlation with the duration of illness. These findings suggested that medicinal drugs or disease duration might be unrelated to the severity of BDP impairment in schizophrenia. Therefore, stereopsis defect in schizophrenia might be state-independent and is not affected by antipsychotic history.

Both duration of illness (Shoshina and Shelepin, 2013), and the type of drug treatment (Shoshina et al., 2014) have been identified as factors that can induce visual contrast sensitivity deficits in patients with schizophrenia, which could further exacerbate cortical visual dysfunction. Our study on FEDN controlled for the effects of illness duration and drug treatments for stereopsis. Impaired BDP in FEDN suggest that stereopsis defects might be state-independent, and there is a moderately strong association between schizophrenia and defective stereopsis.

The generation of a full, stereoscopic depth percept is a multi-stage process that involves both the dorsal and ventral visual information processing streams. Impaired BDP in patients with schizophrenia suggests a potential dysfunction in both the dorsal and ventral cortical pathways (Doniger et al., 2002). Meanwhile, maladaptive neural network connectivity (Uhlhaas and Singer, 2010) and functional synchrony (Yang et al., 2014), as well as cellular and subcellular-level pathophysiology (Glantz and Lewis, 2000; Hashimoto et al., 2008) have been described in patients with schizophrenia at the whole-brain level. Since 3D perception also involves input arising primarily from the magnocellular pathway (Livingstone and Hubel, 1987), and the parvocellular pathway, therefore our results cannot rule out potential dysfunctions in the subcortical pathway and further investigation should be conducted.

Together with existing research (Schechter et al., 2006; Kantrowitz et al., 2009; Hui et al., 2017) on the impairment of stereopsis in patients with schizophrenia, our findings supported the neurodevelopmental hypothesis of schizophrenia. That is, the BDP deficits might develop before the onset of the disease. Generally, stereopsis starts to develop in infancy and fully develops by 9 years old (Aslin and Dumais, 1980). In the current study, a significant disruption in stereo-acuity in both patient groups occurred at 63″ that equate the level of stereo acuity at age 7 in a healthy population (Simons, 1981), which suggests that impaired BDP may occur during childhood. The finding of impaired BDP in FEDN patients provides robust evidence supporting the neurodevelopmental hypothesis for schizophrenia.

Several limitations of this study should be noted. First, a small sample size – particularly for the FEDN group (*n* = 17), which might lead to low statistical power to detect any potential differences with the chronic group. Although the data was shown to have a normal distribution using the normality test, and there were no statistical outliers within any of the three groups according to the mean ± 3σ criterion, we cannot totally rule out the possibility that here are potential differences between FEDN and chronic groups. Second, information on the number of participants wearing glasses during these tests was not collected although we ensure that the VA of all participants should be at least 0.6 (MAR) and the acuity discrepancies between the left and right eye should be no more than 1 line (Snellen chart). Besides, a check of possible confounds from VA and glasses was conducted on another healthy control group. No difference in the BDP between subjects wearing glasses or not and no association between the ocular diopter strength and the BDP threshold was found. The third is referring to the warnings about the possibility to misdiagnosis patients with schizophrenia if relying solely on BDP deficits. This study was both a case-control and between-subject study, therefore the explanation of findings was rather cautious. Some patients with schizophrenia (13/48) had a stereo threshold that was less than 40″ (the norm for healthy adults), and vice versa, there were also a few healthy controls (12/40) with a stereo threshold that was higher than 40″. Whether depth perception examination is of any benefit to diagnosis is still undetermined. Future studies that combine the assessment of stereopsis with an evaluation of early visual measures and having a larger sample size are needed to determine its clinical applicability.

## Conclusion

Patients with schizophrenia exhibited a marked deficiency of stereopsis that is unaffected by drug treatment, and is independent of disease progression. These findings suggest that there was a strong association between stereopsis defect and schizophrenia. However, the neural mechanisms of stereopsis deficits in patients with schizophrenia are still not fully known. Therefore, further ERP/fMRI studies should be conducted to determine the neural mechanisms of stereopsis deficits and whether stereopsis deficits have the potential to be a vital prodromal symptom for schizophrenia.

## Author Contributions

ZW designed behavioral experiments. ZW, ZY, ZP, QZ, KZ, and XW performed the experiments and data analysis. ZW and HWS provided project supervision. XW and DZ provided funds. ZW wrote the paper. XW and ZW revised the manuscript. All the authors discussed and commented on the final version of the manuscript. All the authors critically reviewed content and approved final version for publication.

## Conflict of Interest Statement

The authors declare that the research was conducted in the absence of any commercial or financial relationships that could be construed as a potential conflict of interest.
